# Flaxseed anaphylaxis: an emerging allergen

**DOI:** 10.1097/j.pbj.0000000000000265

**Published:** 2024-10-25

**Authors:** Diogo Mota, Maria João Vasconcelos, Borja Bartolomé-Zavala, Diana Silva, Alice Coimbra

**Affiliations:** a Department of Allergy and Clinical Immunology, São João Hospital, Porto, Portugal; b Unidade de Imunoalergologia, Hospital Lusíadas Porto, Lusíadas Saúde; c R&D Department Roxall, Bilbao, Spain; d Faculty of Medicine, University of Porto - FMUP, Porto, Portugal

## Abstract

Flaxseed is an emerging allergen, and a detailed clinical history is crucial for diagnosis. Flaxseed allergens identified are associated with severe reactions. Providing clear advice on food avoidance might be challenging as there are no reports of clinical cross-reactivity to other foods published.

## Introduction

Flaxseed, also known as linseed, is a seed derived from the flax plant *Linum usitatissimum*. While it is generally considered an uncommon allergen, its relevance is increasingly recognized. Flaxseed is valued for its nutritional content and multiple properties, and its growing consumption in the diet may be responsible for an increasing incidence of food allergy.^1^ Although few cases of flaxseed allergy have been published, they mostly reported severe reactions.^2^

In this report, we aimed to review the published cases of flaxseed-induced anaphylaxis and present two distinct case reports of flaxseed allergy.

## Materials and methods

For the review of the literature, a systematic search was performed in PubMed®/MEDLINE® until February 2024. Only case reports of IgE-mediated flaxseed allergy were found, and all of them were included in this analysis.

The allergic workup included skin prick tests (SPTs) with aeroallergens; skin prick-to-prick tests (SPPTs); and serum-specific IgE (sIgE) to the suspected foods, total IgE, baseline tryptase, ISAC microarray (ThermoFisher®), and SDS-PAGE immunoblotting, performed according to the *Laemmli* method in two electrophoresis conditions: nonreducing (without 2-mercaptoethanol) and reducing (with 2-mercaptoethanol).

## Results

**Figure 1.**
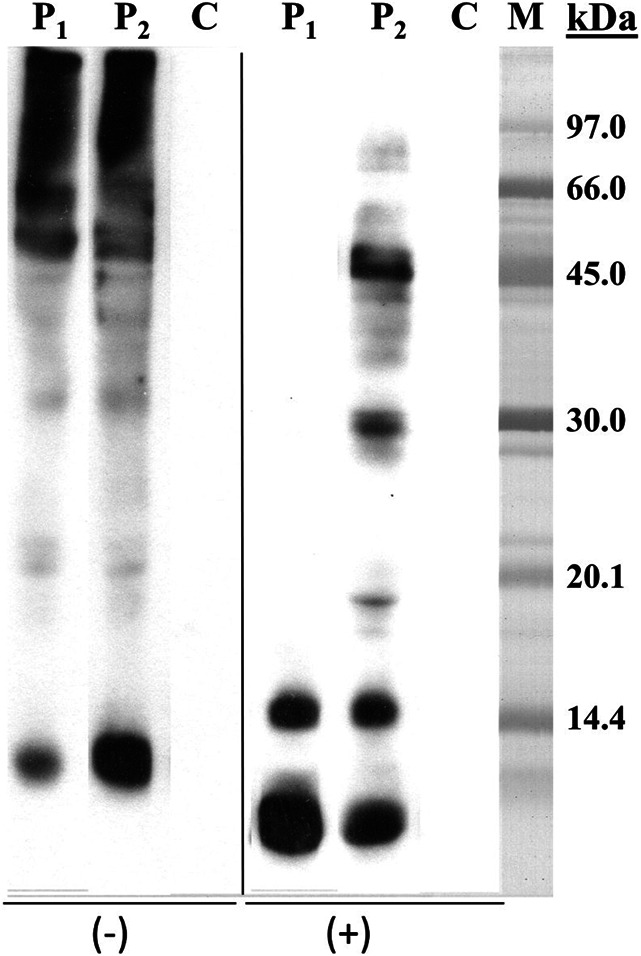
SDS-PAGE immunoblotting. Flaxseed extract. Lane P_1_: case 1 serum, Lane P_2_: case 2 serum, Lane C: control serum (pool of sera from nonatopic subjects), Lane M: molecular mass standard. (−) samples without 2-mercaptoethanol, (+) samples with 2-mercaptoethanol.

The first case is an 81-year-old man, without any history of atopy or food allergies, who ingested a strawberry yogurt with flaxseed powder and papaya. Immediately after, he experienced nausea, emesis, and dyspnea. At admission to the emergency department, he exhibited hypotension, tachycardia, bronchospasm, and generalized maculopapular exanthema. The patient was successfully treated with intramuscular adrenaline, systemic corticosteroids, antihistamines, and nebulized bronchodilators. His acute tryptase level was elevated (17.9 ug/L). After the reaction, he tolerated cow's milk, suggesting flaxseed, papaya, and strawberry as the suspected culprits. Allergic workup was performed 3 months after the reaction and yielded negative SPT results, and SPPT were positive only for flaxseed (11 × 7 mm, histamine 10 mg/mL 5 × 5 mm). Total IgE was 79 kU/L, and flaxseed sIgE was the only positive specific IgE value obtained (18.5 kUA/L). The baseline tryptase level was 5.4 ug/L, and ISAC was negative for all the allergens. SDS-PAGE immunoblotting showed an IgE-reactive band of approximately 13 kDa under nonreducing electrophoresis conditions and a pair of bands of approximately 14.5 kDa and 10 kDa under reducing conditions (Fig. 1). Oral challenge with flaxseed was not performed.

Anaphylaxis to flaxseed was diagnosed and flaxseed avoidance was advised. The patient further tolerated papaya, strawberry, walnut, almond, wheat, and soybean.

The second case is a 9-year-old boy with allergic rhinitis, asthma, and fish allergy who presented with two episodes of urticaria, emesis, abdominal pain, and throat tightness, occurring 5 minutes after consuming yogurt and a milkshake containing flaxseeds. These episodes were treated with systemic corticosteroids and antihistamines at the emergency department. Afterward, he tolerated all the ingredients, except flaxseeds. Allergic workup, conducted 6 months after the reaction, revealed positive SPT results for house dust mites and A*lternaria alternata* and SPPT positive for flaxseed (11 × 5 mm, histamine 10 mg/mL 6 × 5 mm). Total IgE was elevated (1270 kU/L), and sIgE to flaxseed was positive (2.9 kU/L). SDS-PAGE immunoblotting revealed IgE-reactive bands with the same molecular mass as in case 1 and some additional ones (45 kDa, 30 kDa, 19 kDa). ISAC showed multiple sensitizations, including nonspecific lipid transfer protein (nsLTP) (Pru p 3, Jug r 3, and Ara h 9) and storage proteins (Ana o 3, Cor a 8, Jug r 1, and Gly m 6). Oral challenge with flaxseed was not performed.

The patient was advised to avoid consumption of flaxseed, and he subsequently tolerated tree nuts, peanut, wheat, soybean, lupine, and sunflower and sesame seeds.

Flaxseed has the potential to induce severe allergic reactions. We presented two cases of flaxseed-induced anaphylaxis, in patients with different ages and atopic profiles. In our literature review, a total of 13 cases were found, primarily involving middle-aged adults, the majority of them with no history of atopy described (Table 1).^3-14^ Flaxseed allergy was diagnosed based on specific IgE, skin prick-to-prick tests, and other assessments such as immunoblotting. Food challenges with flaxseed were not performed in any case.

**Table 1:** Case reports of flaxseed anaphylaxis.

	Age (yr)/Sex	Atopy	SPPT	sIgE (kU/L)	Other tests	Tolerance of potential cross-reactive foods
Case 1	81/M	No	+	18.5	IB: 13 kDa	Walnut, almond, wheat, soybean
Case 2	9/M	Yes	+	2.8	IB: 13 KDa, 45 kDa, 30 kDa, 19 kDa	Tree nuts, peanuts, wheat, soybean, lupine, sunflower, sesame seeds
Alonso et al (1996)	40/F	No	NA	2.16	SPT+; IB: 38, 35, 30, 22, and 20 kd	
Lezaun et al (1998)	40/M	No	NA	38	SPT+; IB: 35–100 kDa	
León et al (2003)	39/F	Yes	+	20	LHRT positive	
Álvarez-Perea et al (2013)	61/M	NA	+	Positive	IB: 25, 43, 53, 62 kDa	
Lleonart et al (2014)	44/M	Yes	+	2.68	IB: 18 and 20 kDa (oleosin?)	
Koizumi et al (2014)	29/F	NA	+	Positive	BAT positive	
Antolin-Amerigo et al (2016)	50/M	NA	+	NA	IB: 60, 45, 40, 35 and 20 kDa (LTP?)	Fruits and nuts
Kang et al (2017)	42/F	Yes	−	NA	ITD (1/10) +	
Basagaña et al (2018)	46/F	Yes	+	1.25	IB: 60 kDa, 50 kDa, and 12 kDa	
	64/F	Yes	+	2.28	IB: 60 kDa, 50 kDa, 45 kDa, 12 kDa	
Romero-Sanchez et al (2022)	64/M	No	+	NA	IB: 13, 24, and 57 kDa; 58 kDa, 34 kDa, 25 kDa, 16 kDa, 10 kDa, 5 kDa	Walnut, hazelnut, sesame seeds, peanuts, chestnuts, almonds, soybeans
Holbreich et al (2022)	11m/F	NA	−	52.9	NA	
Xavier de Almeida (2023)	1/M	No	+	15.2	NA	Sesame, pumpkin and sunflower seed, pine nut, lentil, black bean, chickpea, green pea, soy

BAT, basophil activation test; F, female; IB, immunoblotting; ITD, intradermal test; LHRT, leukocyte histamine release test; LTP, lipid transfer protein; M, male; NA, data not available; sIgE, serum-specific IgE; SPPT, skin prick-to-prick test; SPT, skin prick test.

## Discussion

Several proteins have been proposed as potential flaxseed allergens, including storage proteins, oleosins, and nsLTP. Leon and Álvarez-Perea identified a 56-kDa allergen, corresponding to malate dehydrogenase-1, as a major allergen in flaxseed.^7,14^ Antolin-Amerigo suggested nsLTP as the relevant allergen in a case of flaxseed anaphylaxis.^6^ In more recent reports (Basagna, 2018, and Romero-Sanchez, 2022), conlinin (Lin u 1), a 2S storage protein, was identified as the culprit allergen, and it was described as a 13-kDa band under nonreducing conditions.^10,11^ In our cases, a protein of approximately 13 kDa was detected under nonreducing conditions and a band of approximately 9 kDa under reducing ones (with 2-mercaptoethanol). The size of these bands also matches the molecular mass published for Lin u 1 (2S storage protein).^2^

Moreover, clinical cross-reactivity among seeds and tree nuts is not well understood, making it challenging to provide clear advice on food avoidance. Possible cross-reaction with other foods have been described, namely lupine, peanut, rapeseed, soybean, wheat, and rape pollen.^1^

While in vitro storage protein cross-reactivity seems high, in vivo reactions may primarily occur within the same botanical family. Importantly, none of the previous reports mentioned reactions to other seeds or tree nuts, although no oral challenges were performed. In our second case, the patient tolerated many other tree nuts and seeds, despite showing multiple sensitizations in ISAC, including other storage proteins.

Identifying the proteins responsible for eliciting reactions remains a considerable challenge, as allergens are often present in small amounts or as hidden components. A comprehensive clinical history is crucial for diagnosis since flaxseed is frequently overlooked as a potential allergenic source during allergic workups. In addition, multiplex immunoassays (ImmunoCAP ISAC, Thermo Fisher Scientific, Sweden, and ALEX Allergy Explorer Macro Array Diagnostics GmbH, Austria), often used for identifying potential allergens in patients experiencing unexplained anaphylaxis, do not include flaxseed.

Furthermore, given the unclear patterns of clinical cross-reactivity, flaxseed-allergic patients may be advised about the potential risk of reaction to other seeds or tree nuts. A shared decision on whether to pursue further investigation should be made, and patients may be recommended to carry an emergency plan.
